# Effects of a myosin light chain kinase inhibitor on the optics and accommodation of the avian crystalline lens

**Published:** 2011-10-22

**Authors:** Sara Luck, Vivian Choh

**Affiliations:** School of Optometry, University of Waterloo, Ontario, Canada

## Abstract

**Purpose:**

While many studies investigate the cytoskeletal properties of the lens with respect to cataract development, examinations of how these molecular structures interact are few. Myosin light chain kinase (MLCK), actin, and myosin are present on the crystalline lenses of chickens. The purpose of this experiment was to determine whether contractile proteins found on the lens play a role in the optical functions of the lens at rest, and during accommodation.

**Methods:**

Eyes of 6-day old white Leghorn chicks (*Gallus gallus domesticus*) were enucleated, with the ciliary nerve intact. One eye was treated with the MLCK inhibitor 1-(5-iodonaphthalene-1-sulfonyl)-1H-hexahydro-1,4-diazepine hydrochloride (ML-7) and the other eye with vehicle only. Three concentrations of ML-7 were used: 1 µM, 10 µM, and 100 µM. The back vertex focal lengths (BVFLs) were measured before, during, and after accommodation using an optical laser scanning monitor (Scantox™). To further confirm ML-7 activity, western blotting was performed to detect whether MLCK was inhibited.

**Results:**

Western blots confirmed that MLCK was inhibited at all three ML-7 concentrations. Ten µM ML-7 treatments led to longer BVFLs at rest (p=0.0338), while 100 µM treatments led to opposite changes, resulting in shorter BVFLs (p=0.0220). While 1 µM treatments did not lead to significant optical changes (p=0.4416), BVFLs were similar in pattern to those of the 10 µM group. ML-7 had no effects on accommodative amplitudes (p=0.7848).

**Conclusions:**

Inhibition of MLCK by ML-7 led to differential changes in BVFLs that presumably affected lenticular integrity. No apparent effect on accommodative amplitudes was observed.

## Introduction

Accommodation in humans was first described by von Helmholtz as a cascade of events involving ciliary muscle contraction, reduction in the tension of the zonules connecting the ciliary muscle and the lens, and finally, a change of the lens shape so that its surfaces are more curved, resulting in a higher refractive power [1]. The crux of this model is that the lens is pliable, and as such, undergoes mechanical stress during accommodation.

Motility and stress of a cell involve cytoskeletal components such as intermediate filaments, actin, myosin, and adhesion proteins such as the cadherins. While many studies investigate the cytoskeletal properties of the lens with respect to cataract development, very little information pertaining to how these molecular structures interact, or are altered during accommodation, exists. Previous studies revealed a network of filamentous f-actin polygonal arrays that are colocalized with myosin in the anterior epithelium of the lens [2,3]. A similar arrangement of actin and myosin was observed at the posterior surface on the capsule in chicken lenses, along with other proteins, such as N-cadherin, myosin light chain kinase (MLCK), and additional proteins that are involved in contraction [4]. These findings may suggest that lenticular forces could contribute to accommodation. Cellular movements are known to occur when there is a presence of both actin and myosin; their interactions form the basis of a molecular motor, and this motor is found in muscle tissue as well as in non-muscle tissue.

The purpose of this study was to determine whether the contractile proteins found on the lens play a role in the optical functions of the lens at rest, and during accommodation. Since MLCK is found in lenticular cells, an MLCK inhibitor, such as 1-(5-iodonaphthalene-1-sulfonyl)-1H-hexahydro-1,4-diazepine hydrochloride (ML-7), would be expected to disrupt the cytoskeletal proteins on the lens, such as actin and myosin. It is known that phosphorylation of MLCK leads to various physiological processes, including contraction of smooth muscle, fibroblast contraction as well as cytoskeletal modeling by (actin) stress fibers in nonmuscle cells [5,6]. Therefore, inhibiting MLCK on the lens would interfere with the possible contraction that is taking place, resulting in a change in accommodative amplitude.

## Methods

### Eye dissections and lens treatments

White leghorn hatchling chicks (*Gallus gallus domesticus*) were obtained from Maple Leaf Poultry, New Hamburg, Ontario, Canada and were fed ab libitum with lights on a 14:10 light: dark cycle. Chicks were cared for in accordance with the guidelines of the Canadian Council on Animal Care; their management is in accordance with guidelines established by the Institute for Laboratory Animal Research.

Chicks were sacrificed by decapitation when they were 6 days old. Heads were bisected along the sagittal plane. Eyes were enucleated and the posterior globe was removed except for a wedge containing the intact ciliary nerve and ganglion. For optical function assessments, the sclera was removed as close to the lens as possible without damaging the ciliary muscle so that the lens could be viewed by the cameras located in the Scantox™ In Vitro Lens Assay System (XTOX Scientific, Napean, ON). For the western blot procedure, the vitreous was also removed before removal of the posterior portion of the lens capsule. All dissections were performed in oxygenated (95% oxygen, 5% carbon dioxide) Tyrode’s saline (TS: 134 mM NaCl, 3 mM KCl, 20.5 mM NaHCO_3_, 1 mM MgCl_2_, 3 mM CaCl_2_, in deionized water).

For all experiments, either the left or the right eye of each pair was treated for 15 min with 1 µM ML-7 in 0.001% (v/v) ethanol (EtOH) in TS, 10 µM ML-7 in 0.01% (v/v) EtOH in TS or 100 µM ML-7 in 0.1% (v/v) EtOH in TS while the fellow eyes of each pair was treated with the appropriate concentration of vehicle (0.001% (v/v) EtOH in TS, 0.01% (v/v) EtOH in TS or 0.1% (v/v) EtOH TS, respectively).

### Measurements of lenticular optical function

Following either ML-7 or vehicle treatment, each eye was pinned to a Sylgard^®^ (Dow Corning, Midland, MI) washer, with the cornea facing down. The eye was then placed into a glass chamber with an access tube. A custom-made suction electrode, connected to a Grass S43 stimulator (Astro-Med, Inc., Brossard, QC), was passed through the access tube before plugging the rest of the small tube with petroleum jelly. The chamber was filled with 20% (v/v) fetal bovine serum (PAA Laboratories, Etobicoke, ON) in TS to visualize the refracted beams. The ciliary nerve was suctioned into the electrode tip.

Lenses were scanned using the Scantox™ In Vitro Lens Assay System (XTOX Scientific) as previously described [7,8]. In brief, a low-powered helium laser passed through the lenses at 0.131 mm intervals away from the optical axis and the back vertex focal lengths (BVFLs), the distances from the back vertex of the lens to where refracted beams cross the optical axis, were measured using the Scantox™ system. Scans were obtained before, during and after accommodation. Accommodation of the intact eye was induced by electrical stimulation of the ciliary nerve (30 Hz, 1–1.5 mA).

### Statistical analysis of measurements of optical function

For each scan, the three central BVFLs were removed because of the presence of sutures, regions of optical disorder that can over- or under-estimate back vertex focal lengths. For each pair of eyes, the scan with the smallest number of beams was the standard for all other scans for that chick. BVFLs were converted from millimeters into dioptric values using the refractive index of water, n=1.33. Accommodative amplitude was calculated as the difference between the mean pre-stimulation and stimulation vergence values for each eye. Optical quality of the lens was assessed using two parameters, focal length variability, which increases the amount of scatter due to optical degradation, and spherical aberration (SA), which is a measure of the deviation of marginal rays from those at the optical axis.

Mixed model repeated measures analysis of variance (ANOVA) tests were used to determine changes in back vertex focal lengths (BVFLs), focal length variability and spherical aberration (SA) as a function of inhibition treatments (ML-7 versus vehicle), dosage groups (1 µM, 10 µM, and 100 µM) and accommodative state (pre-stimulation, stimulation and post-stimulation). Changes associated with accommodative amplitude were also assessed using a mixed model repeated measures ANOVA as a function of inhibition treatments and dosage group. One-way repeated measures ANOVAs with Bonferroni-corrected multiple comparison tests were used as post-hoc tests for further analysis of interactions between dependent or repeated data. Greenhouse-Geisser correction factors were used for epsilon <0.75. Results were significant at p≤0.05.

### Western blot analysis

Western blots of the relative amounts of phosphorylated myosin in the presence of ML-7 and its vehicle (ethanol; EtOH) were used to confirm that ML-7 had an inhibitory effect on myosin light chain kinase activity. The posterior capsule of the lenses was removed then placed in cold 20 µl of Phosphate-Buffered Saline (PBS: 137 mM NaCl, 3 mM KCl, 101 mM Na_2_HPO_4_, 2 mM KH_2_PO_4_, in deionized water). Capsule samples were then centrifuged (3,786× g for 10 min at 4 °C) and the supernatant collected. The total protein concentration of each sample was quantified using the BioRad DC protein assay™ (500–0111; BioRad Laboratories, Mississauga, ON). Capsule samples were mixed with Biostab (62513; Biomol, Hamburg, Germany), a biomolecular storage solution (5:1 ratio, respectively) before storing at –80 °C.

Capsule samples were diluted with gel loading buffer (GLB: 100 mM Tris base [pH 7.4], 5% [v/v] glycerol, 2.5% [v/v] SDS, 0.03% [w/v] bromophenol blue, in water) then heated (3 min at 95 °C). For each western blot, samples (1 µl) were run at the same concentrations to ensure equal loading of protein. Concentrations below 1 µg/µl were not used. Samples were loaded on a polyacrylamide gel (PhastGel™, gradient 10–15, 57–6781–00; GE Healthcare Bio-Sciences, Mississauga, ON) and run on a GE Phastsytem™ Separation & Control Unit (GE Healthcare, Uppsala, Sweden, at 250 V, 15 °C, 30 min) with SDS Buffer strips (17–0515–01; GE Healthcare Bio-Sciences). Proteins were electro-transferred onto wet immuno-blot polyvinylidene-difluoride (PVDF) membranes (162–0176; BioRad Laboratories) and membranes were dried (RT, 15 min followed by 50 °C at 45 min).

Membranes were blocked (blocking buffer [BB]: 8% [w/v] skim milk powder in 0.5% [v/v] Tween-20 in PBS [PBS-T], 45 min) then washed in PBS-T (5 min) before submerging in anti-phospho-myosin (M6068; Sigma, St. Louis, MO; 1:1000 in 90% PBS-T: 10% BB, 4 °C overnight). Membranes were then washed (4×5 min, PBS-T) and anti-phospho-myosin was labeled with horseradish peroxidase-conjugated goat anti-rabbit IgG solution (H & L, ab6721; Abcam, Cambridge, MA; 1:1000 in 90% PBS-T: 10% BB; 1 h, RT). Membranes were washed (3×5 min, PBS-T) followed by a final wash (PBS, 1 min). Proteins were visualized with chemiluminescent ECL™ detection (RPN2132, RPN2133; Amersham Biosciences, Mississauga, ON) and imaged using the Storm 840™ imaging system (Molecular Dynamics, Sunnyvale, CA). Protein expression was measured by quantifying the amount of fluorescence using the ImageQuant™ software.

## Results

### Measurements of optical functions of the lens

**Figure 1:**
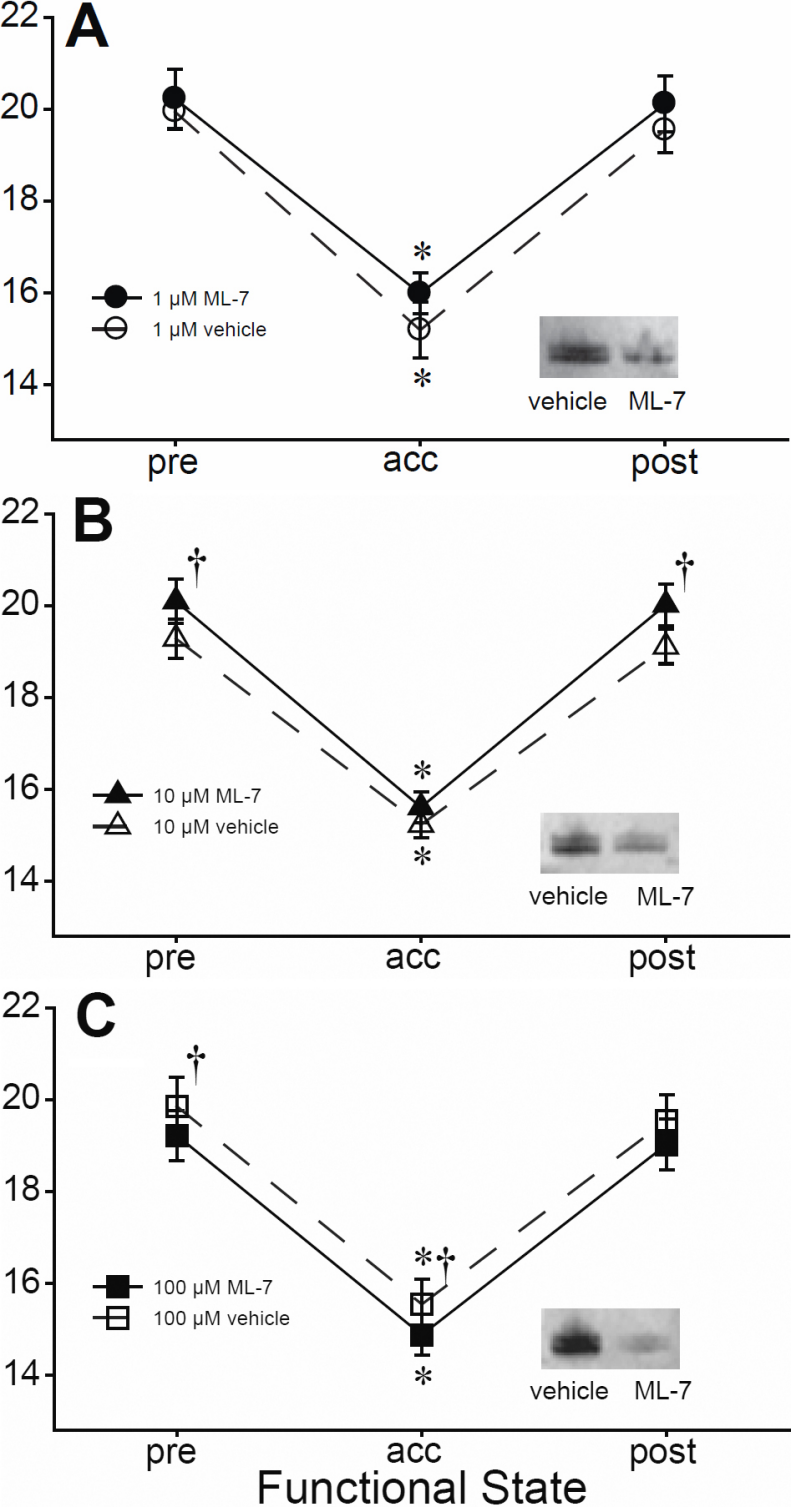
Effects of ML-7 on mean back vertex focal lengths (±s.e.m). Line graphs show the effects of lenses treated with ML-7 (filled symbols) and vehicle (empty symbols) pre-, during, and post-accommodation. **A**: 1 µM (n=4), **B**; 10 µM (n=12), **C**: 100 µM (n=9). For all three dosage groups, focal lengths for the accommodation state were significantly shorter than those at rest (p<0.0001; denoted by asterisks). Daggers denote significant differences in BVFLs between ML-7- and vehicle-treated eyes at the same functional state. Insets: western blots showing relative amounts of phosphorylated myosin for all dosage groups. Please refer to the text for relative intensity values.

As expected, differences in the mean back vertex focal lengths (BVFLs) were detected as a function of accommodative state, with BVFLs during accommodation shorter than those at rest for all three dosage groups (p<0.0001; Figure 1). ML-7 at all doses inhibited MLCK activity, as shown by the relatively lower amounts of phosphorylated myosin in western blots of the lenticular posterior capsules treated (intensity relative to respective vehicle: 1 µM 50.7%; 10 µM 47.2%; 100 µM:16.7%; Figure 1: insets), but did not have an overall (main) effect on BVFLs (Figure 1). However, interaction between dosage group and inhibition treatments was detected (p=0.0138), indicating that ML-7 had differential effects on BVFLs. Post-hoc tests indicated that both the 10 µM and 100 µM ML-7 treatments had effects on BVFLs (p=0.0338 and p=0.0220, respectively) that were opposite. For the 10 µM group, BVFLs of ML-7-treated eyes were significantly longer than those for vehicle-treated eyes during the two resting states (Figure 1B; pre-accommodation mean±s.d.: 20.10±1.65 mm versus 19.28±1.48 mm, respectively, p=0.0027; post-accommodation: 20.02±1.57 mm versus 19.10±1.29 mm, p=0.0015), but not during accommodation (15.61±1.18 mm versus 15.23±1.02 mm, p=0.6477; Figure 1B). While differences in mean BVFLs for the 1 µM group were not significant (Figure 1A; p=0.4416), BVFLs were similar in pattern to those of the 10 µM group, with ML-7-treated eyes showing slightly, although not significantly longer BVFLs than their fellow vehicle-treated eyes (pre-accommodation: 20.22±1.44 mm versus 19.94±0.85 mm, respectively, p=1.000; accommodation: 15.98±1.00 mm versus 15.19±1.35 mm, p=0.4623; post-accommodation: 20.11±1.35 mm versus 19.54±1.11 mm, p=1.000, respectively; Bonferroni; Figure 1A). ML-7 treatment (100 µM) resulted in the opposite trend to the two lower doses, with ML-7-treated eyes showing shorter BVFLs compared to their fellow control eyes (Figure 1C; pre-accommodation: 19.22±1.64 mm versus 19.85±1.90 mm, p=0.0132; accommodation: 14.83±1.34 mm versus 15.54±1.65 mm, p=0.0082; post-accommodation: 19.02±1.66 mm versus 19.54±1.66 mm, p=0.0615).

### Measurements of accommodation of the lens

**Figure 2:**
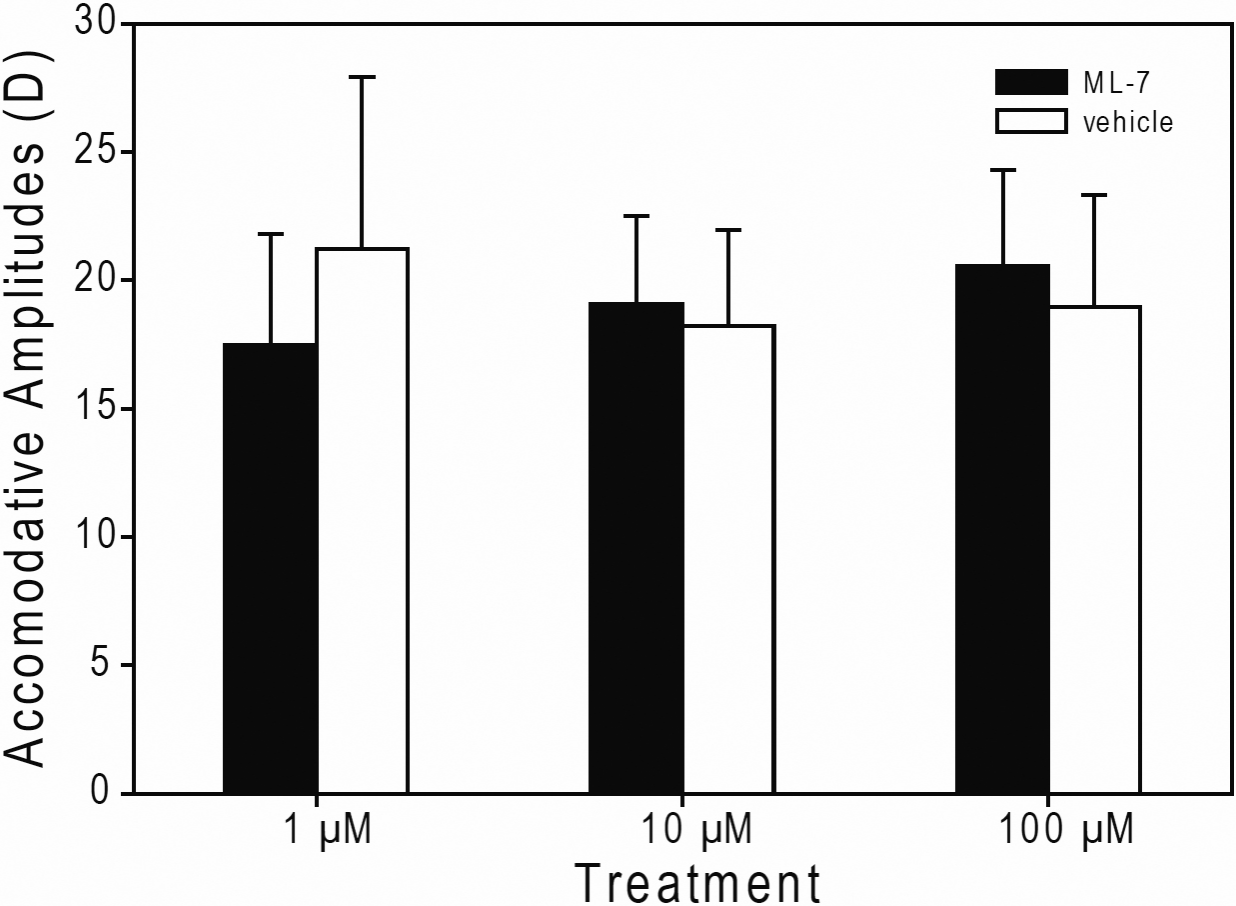
Effects of ML-7 on accommodative amplitudes (±s.e.m). Bar graphs show the accommodative amplitudes of 1 µM (n=4), 10 µM (n=12), and 100 µM (n=9) ML-7-treated (filled bars) and vehicle-treated (empty bars) eyes, respectively.

Although ML-7-induced differences in BVFLs were detected for pre-stimulation states for the 10 µM and 100 µM ML-7 treatment groups, accommodative amplitudes were not different across dosage groups (p=0.7848), inhibition treatments (p=0.6112), nor was any interaction detected between these two factors (p=0.0646; Figure 2).

## Discussion

### Effects of ML-7 on lenticular optics

This study was performed to determine whether contractile microfilaments could affect the optics or accommodative amplitudes of the lens. The data indicated that ML-7 has effects on the optics of the lens, resulting in longer mean BVFLs for lower doses of ML-7 in eyes at rest. The lower BVFLs indicate a change in the shape of the lens, presumably a flattening, which could arise as a result of a loss of tone or stiffness of the lens or of the ciliary muscles that surround and directly articulate the lens. Ciliary muscle in chicks is skeletal in nature and is therefore likely unaffected by ML-7; since ML-7 preferentially inhibits smooth muscle MLCK (smMLCK) [9-11], and given that smMLCK expression is lower than that of skeletal MLCK in skeletal muscles [12], we speculate that ML-7 effects on ciliary muscle, if any, would be negligible. Moreover, unpublished data from our laboratory indicate that isolated chicken lenses exposed to low concentrations of ML-7 become softer (as assessed by lower amounts of force exerted by the lens upon being compressed between two metal plates in solution; data not shown), which, together with the observations that ML-7 had no effect on accommodative amplitudes from contraction of the ciliary muscles (Figure 2), suggest that ML-7 acted directly via changes to lenticular integrity and consequently, lenticular shape, rather than via the ciliary muscle. Our western blots of the posterior lens capsule confirming that ML-7 treatments were effective in reducing the phosphorylation of myosin (Figure 1, insets) would seem to further support a role for the lens, and moreover, that loss of stiffnes was a result of ML-7-induced inhibition of MLCK and its subsequent effects on myosin and actin at the lens capsule. Although we did not carry out experiments to confirm whether myosin and actin distributions in the lenses were altered with ML-7, other investigators have shown that ML-7 reduces the stiffness of nonmuscle cells such as fibroblasts [13], and induces loss of, or prevents formation of actin stress fiber bundles in human carcinoma [14], fibroblast [14-16] and endothelial cells [17,18]. A study by Collin and colleagues [19] showing that relaxation of baby hamster kidney cell podosomes is associated with the disappearance of actin stress fibers adds support to our findings that ML-7-induced loss of stiffness is almost certainly related to loss of actin stress fiber formation.

The possibility that changes in BVFLs were a result of changes to the refractive index of the lens must also be acknowledged. Presumably, for such a decrease in refractive index to occur, and within the time span observed, ML-7 would have to directly affect the crystallin proteins. However, we do not believe that this could occur without causing changes to the optical quality of the lenses and both the focal length variability and the negative spherical aberrations that are typical of avian lenses were observed in our experiments to be unaffected by ML-7 (data not shown). We know of no mechanism for ML-7 to affect the down-regulation of the crystallin proteins.

That treatment of 100 µM ML-7 results in the opposite response, i.e., shorter, rather than longer BVFLs, would suggest that the cytoskeletal integrity and stiffness of the lenses were also opposite to lenses treated with lower doses of ML-7. Cytoskeletal distributions were not examined in this study but preliminary results of a parallel study by our group indicate that a 100 µM dose of ML-7 leads to stiffer (isolated) chick lenses [20]. A dual or biphasic response for ML-7 in other nonmuscle cells was reported by Takizawa and colleagues [14], who showed that cell-spreading behaviors were opposite depending on the the dose of ML-7. That BVFLs of lenses were diametrical in our study might be related to the specificity of ML-7 and its mode of inhibition. At higher doses, ML-7 inhibits kinases other than MLCK, such as protein kinase A (PKA) and protein kinase C (PKC; K_i_ values of 21 µM and 42 µM, respectively; [21]). Of the two kinases, PKA is the likelier candidate as an enzyme whose inhibition could lead to opposite effects on cytoskeletal integrity and therefore by inference, on the stiffness of the lens and BVFLs. Microinjection of PKA into living fibroblast cells leads to PKA-induced dephosphorylation of MLCK and actin filament disassembly [22], while inhibition of PKA activity in endothelial cells results in the opposite, i.e., actin stress fiber formation [23]. However, a role for PKA remains uncertain as it assumes that the effects of PKA inhibition are stronger than those of MLCK inhibition, which is not known. It should also be noted that opposing behaviors may not be related to secondary inhibition effects of ML-7, but rather to other regulatory proteins that differentially mediate the sensitivities of the cytoskeletal components to ML-7 [14].

As observed in previous experiments [7,8], optical scans for all lenses showed negative spherical aberrations (SAs), which increased with accommodation (p=0.0194; data not shown), although no changes in SA were detected as a function of ML-7 treatment (p=0.1933; data not shown). These results suggest ML-7 effects were similar across the entire anterior and posterior surfaces of the lens; observations that BVFLs were longer with ML-7 are also consistent with the idea of a global effect of ML-7 on the lens.

### Effect of ML-7 on accommodation

Unlike a previous study, which showed that different resting state BVFLs could have effects on accommodative amplitudes [7], no changes in accommodative amplitudes were observed with ML-7 treatment (Figure 2), despite ML-7-dependent differences in resting state BVFLs (Figure 1). Differences in the accommodative amplitudes arising from changes in lenticular integrity may have been too subtle to detect. Alternatively, contributions by the lens may have been masked by the contractile forces applied by the ciliary muscle; ciliary muscle had about a 10-fold larger contractile force across all groups (~20 D, Figure 2) compared to the equivalent change in power between ML-7-treated and vehicle-treated eyes at rest (~2 D, calculated from BVFL vergences, calculation not shown).

In summary, inhibition of MLCK led to changes in the optics of the lens, resulting in a change in BVFL, and therefore presumably, to lenticular shape and integrity. However, putative integrity changes were not reflected in the accommodative amplitudes observed, which may have been a consequence of masking effects by larger ciliary muscle contractions. Thus, we could not unequivocally rule out a role for contractile proteins in accommodation. A preliminary study is planned to determine whether lenticular integrity changes arising from alterations to cytoskeletal proteins can affect accommodation.
